# Supplementary material for: Biodegradation of Free Gossypol by Helicoverpa armigera Carboxylesterase Expressed in Pichia pastoris
Source: Toxins (Basel). 2022 Nov 22;14(12):816. doi: 10.3390/toxins14120816 (PMC9788223; doi:10.3390/toxins14120816)
Supplement: Supplementary file 1 [file toxins-14-00816-s001.zip › toxins-1954049-supplementary.pdf]

toxins

Supplementary Materials

Biodegradation of Free Gossypol by *Helicoverpa armigera* Carboxylesterase expressed in *Pichia pastoris*

Li Zhang, Xiaolong Yang, Rongzheng Huang, Cunxi Nie, Junli Niu, Cheng Chen and Wenju Zhang

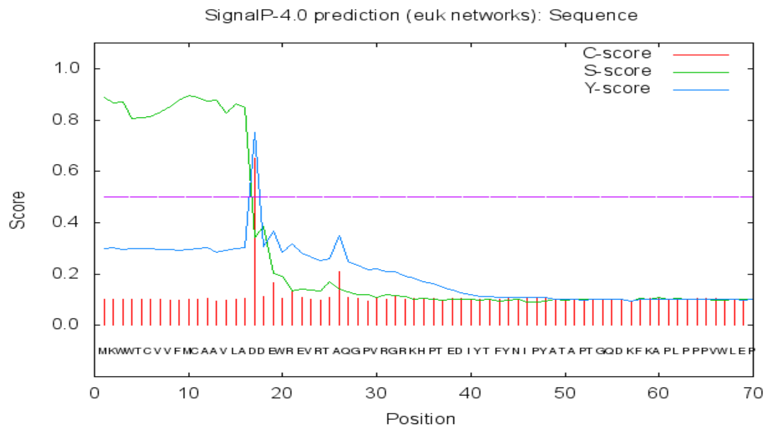

Figure S1. CCE001a has a signal peptide at the 17 amino acid position.

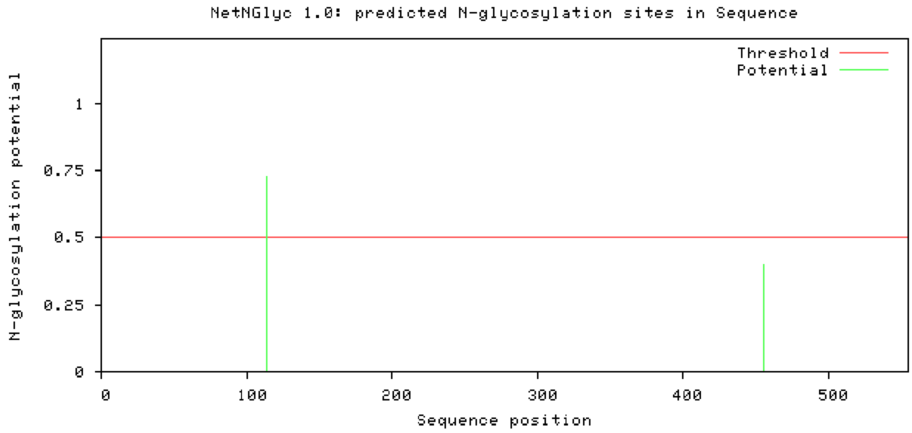

Figure S2. CCE001a protein contains an N-glycosylation site. The protein has an N-glycosylation site.

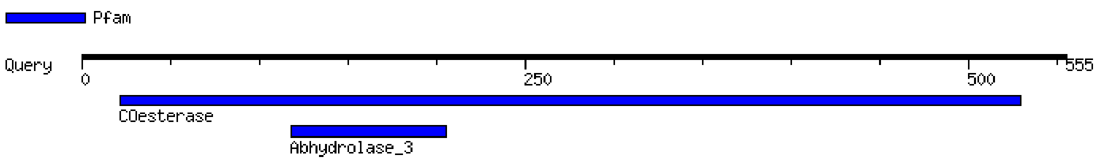

Figure S3. CCE001a protein showed a co-esterase family (PF00135) domain and a dehydrogenase family (PF07859) domain.

Data file S1. *cce001a* DNA sequence:

atgaagtggt ggacgtgtgt ggtgttcatg tgcgcggccg tgctggctga cgacgagtgg

61 cgcgaggtga ggactgcgca agggccggtg cgcgggcgca agcaccccac tgaagatata

121 tacaccttct acaacatacc ctacgccacc gcgcccacgg gccaggataa gttcaaggca

181 cctcttccgc caccagtgtg gttagaacca tttgacgcag tcgacgagca cgttatatgc

241 ccacagccaa tgtttcctgg tgatctcatg cccacaaatg tagtgaccaa agaaaattgt

301 ctcatcgcca acgtatttgt gcctaataca aaagaaaaga acctttcagt tgtcgtatat

361 gtacatggag gagcttttat tatgggctgg ggggaaatgt ttaaggcaag acaattcatg

421 aagacaaaag attttattgt ggtgacgttt aattaccgcc ttggaattca cgggttccta

481tgtctgggca ctgaggacgc gccaggcaat gctggcatga aagaccaggt ggcgctgctg

541 cgctgggtgc agaagaacat cgccagcttc ggcggtaacc ctgatgatgt tactattgca

601 gggtatagtg caggttccgc atcagtagat ctgttaatgc tttcgaaatc agccgaaggg

661 ttatttcacc gagttatacc agaaagtggc ggaaatcttg ccgcattttc aattcaacgg

721 gatcctgtcg agattgctaa atcatacgcc agcaaattag gcttcgataa cggagacgat

781 atttatgcgt tagggaagtt ttatatgaca gctccaattg aaaagttgac gtccgatccg

841 ttttttgaca gaactgattc tacatttttg ttcgcaccat gtgtagaacg tgaaacaggg

901 gacggagcct tcctgactga atcacctcta acaattctaa agactggcaa ctacagaaag

961ctgccagtgt tgtatggatt cgctgaaatg gaaggattaa tacgtattga tttctttgaa

1021 ctttggaagc ataaaatgaa tgaaaagttt tctgatttct tgccagctga cttgaaattt

1081 gattcagaag aagaaagaga agaagtggca aataagataa aggagtttta ctttggcgac

1141 aagccagtcg gcaatgaaaa cattttaaaa tacgttgatt tcttttcgga tgttatattt

1201 gcttacccca tgctttgggc tgtgaagcta cacgtcgaag ctggtaacaa tcaagtatat

1261 ttgtatgaat atagctttgt ggacgaagat gttcctgtgg tacctcatac taatatacgt

1321 ggagctaacc actgtgccca gactatggct ttgagtgatg ggaaaaactt cacacaccat

1381 gatgacaccc tcgcaacacc acagtttaga gaaatgaaaa agactattcg tgaaatatgg

1441cacaattttg tgaaaactgg agtgccagtg ccagagggct catggctgcc ggcgtggccg

1501 gcggcgggcg cggaccgggc gccgcacatg tcgctgggcg agcggctgga gctgcgcggc

1561 gcgctgctgg cggagcgcac gcgcttctgg gatgacatct accagagata ctaccgggac

1621 gcggtgccgc cgcccacacc cccgcccaga ccacgagacg agttgtag

CCE001a protein sequence:

MKWWTCVVFMCAAVLADDEWREVRTAQGPVRGRKHPTEDIYTFY

NIPYATAPTGQDKFKAPLPPPVWLEPFDAVDEHVICPQPMFPGDLMPTNVVTKENCLI

ANVFVPNTKEKNLSVVVYVHGGAFIMGWGEMFKARQFMKTKDFIVVTFNYRLGIHGFL

CLGTEDAPGNAGMKDQVALLRWVQKNIASFGGNPDDVTIAGYSAGSASVDLLMLSKSA

EGLFHRVIPESGGNLAAFSIQRDPVEIAKSYASKLGFDNGDDIYALGKFYMTAPIEKL

TSDPFFDRTDSTFLFAPCVERETGDGAFLTESPLTILKTGNYRKLPVLYGFAEMEGLI

RIDFFELWKHKMNEKFSDFLPADLKFDSEEEREEVANKIKEFYFGDKPVGNENILKYV

DFFSDVIFAYPMLWAVKLHVEAGNNQVYLYEYSFVDEDVPVVPHTNIRGANHCAQTMA
LSDGKNFTHHDDTLATPQFREMKKTIREIWHNFVKTGVPVPEGSWLPAWPAAGADRAP
HMSLGERLELRGALLAERTRFWDDIYQRYYRDAPPPPTPPPRPRDEL

Note:The signal peptide sequence is highlighted in yellow

pPICZαa-CCE001a sequence:

AGATCTAACATCCAAAGACGAAAGGTTGAATGAAACCTTTTTGCCATCCGACATCCACAGGTCCATT
CTCACACATAAGTGCCAAACGCAACAGGAGGGGATACACTAGCAGCAGACCGTTGCAAACGCAGGA
CCTCCACTCCTCTTCTCCTCAACACCCACTTTTGCCATCGAAAAACCAGCCCAGTTATTGGGCTTGAT
TGGAGCTCGCTCATTCCAATTCCTTCTATTAGGCTACTAACACCATGACTTTATTAGCCTGTCTATCCT
GGCCCCCCTGGCGAGGTTCATGTTTGTTTATTTCCGAATGCAACAAGCTCCGCATTACACCCGAACAT
CACTCCAGATGAGGGCTTTCTGAGTGTGGGGTCAAATAGTTTCATGTTCCCCAAATGGCCCAAAACT
GACAGTTTAAACGCTGTCTTGGAACCTAATATGACAAAAGCGTGATCTCATCCAAGATGAACTAAGT
TTGGTTCGTTGAAATGCTAACGGCCAGTTGGTCAAAAAGAAACTTCCAAAAGTCGGCATACCGTTTG
TCTTGTTTGGTATTGATTGACGAATGCTCAAAAATAATCTCATTAATGCTTAGCGCAGTCTCTCTATC
GCTTCTGAACCCCGGTGCACCTGTGCCGAAACGCAAATGGGGAAACACCCGCTTTTTGGATGATTAT
GCATTGTCTCCACATTGTATGCTTCCAAGATTCTGGTGGGAATACTGCTGATAGCCTAACGTTCATGA
TCAAAATTTAACTGTTCTAACCCCTACTTGACAGCAATATATAAACAGAAGGAAGCTGCCCTGTCTTA
AACCTTTTTTTTTATCATCATTATTAGCTTACTTTCATAATTGCGACTGGTTCCAATTGACAAGCTTTT
GATTTTAACGACTTTTAACGACAACTTGAGAAGATCAAAAAACAACTAATTATTCGAAACGATGAGA
TTTCCTTCAATTTTTACTGCTGTTTTATTCGCAGCATCCTCCGCATTAGCTGCTCCAGTCAACACTACA
ACAGAAGATGAAACGGCACAAATTCCGGCTGAAGCTGTCATCGGTTACTCAGATTTAGAAGGGGATT
TCGATGTTGCTGTTTTGCCATTTTCCAACAGCACAAATAACGGGTTATTGTTTATAAATACTACTATT
GCCAGCATTGCTGCTAAAGAAGAAGGGGTATCTCTCGAGAAAAGAGAGGCTGAAGCTGAATTCATG
AAGTGGTGGACTTGTGTTGTTTTTGCTTGTGCTGCTGTTTTGGCTGATGATGAATGGAGAGAAGTTAG
AACTGCTCAAGGACCAGTTAGAGGTAGAAAGCATCCTACTGCTGATATGTACGCTTTTTACAACATT
CCTTACGCTACTGCTCCAACTGGTCAAGATAAGTTTAAGGCTCCATTGCCACCTCCAGTTTGGTTGGA
ACCATTTGATGCTATTGATGAACATGTTATTTGTCCACAACCAATGTTTCCAGGTGATTTGATGCCTA
AGAACGTTGTTACTAAGGAAAACTGTTTGATTGCTAACGTTTTTATGCCAAACACTAAGGAAAAGAA
CTTGTCCGTTGTTGTTTACGTTCATGGTGGAGCTTTTATTATGGGATGGGGTGAAATGTTTAAGGCTA
GACAATTTATGAAAACTAAGGATTTTATTGTTGTTACTTTTAACTACAGATTGGGTATTCATGGATTT
TTGTGTTTGGGAACTGATGATGCTCCAGGTAACGCTGGTATGAAGGATCAAGTTGCTTTGTTGAGATG
GGTTCAAAAGAACATTGCTTCCTTTGGAGGTAACCCAGATGATGTTACTATTGCTGGATACTCAGCTG
GATCAGCTTCCGTTGATTTGTTGATGTTGTCAAAGTCCGCTGAAGGATTGTTTCATAGAGTTATTCCT
GAATCCGGAGGTAACTTGGCTGCTTTTTCTATTCAAAGAGATCCAGTTGAAATTGCTAAGTCATACGC
TTCTAAGTTGGGATTTGATAACGGAGATGATATTTACGCTTTGGGTAAGTTTTACATGACTGCTCCTA
TTGAAAAGTTGACTTCCGATCCATTTTTTGATAGAACTGATTCTACTTTTTTGTTTGCTCCATGTGTTG
AAAGAGAAACTGGTGATGGAGCTTTTTTGACTGAATCCCCATTGACTATTTTAAAGACTGGTAACTA
CAGAAAGTTGCCTGTTTTGTACGGATTTGCTGAAATGGAAGGATTGATTAGAATTGATTTTTTTGAAT
TGTGGAAGCATAAGATGAACGAAAAGTTTTCCGATTTTTTGCCAGCTGATTTGAAGTTTGATTCCGAA
GAAGAAAGAGAAGAAGTTGCTAACAAGATTAAGGAATTTTACTTTGGAGATAAGCCAGTTGGTAAC
GAAAACATTTTGAAGTACGTTGATTTTTTTTCAGATGTTATTTTTGCTTACCCAATGTTGTGGGCTGTT
AAGTTGCATGTTGAAGCTGGTAACAACCAAGTTTACTTGTACGAATACTCCTTTGTTGATGAAGATGT
TCCAGTTGTTCCTCATACTAACATTAGAGGAGCTAACCATTGTGCTCAAACTATGGCTTTGTCCGATG
GTAAGAACTTTACTCATCATGATGATACTTTGGCTACTCCACAATTTAGAGAAATGAAAAAAACTAT

TAGAGAAATTTGGCATAACTTTGTTAAGACTGGAGTTCCAGTTCCTGAAGGTTCATGGTTGCCAGCTT
GGCCAGCTGCTGGAGCTGATCGCGCGCCTCACATGTCCTTGGGTGAAAGATTGGAATTGAGAGGAGC
TTTGTTGCCTGAAAGAACTAGATTTTGGGATGATATTTACCAAAGATACTACAGAGATGCTGTTCCAC
CACCTAAGCCACCACCTAGACCTAGAAACGAATTGTTTCTAGAACAAAAACTCATCTCAGAAGAGGA
TCTGAATAGCGCCGTCGACCATCATCATCATCATCATTGAGTTTGTAGCCTTAGACATGACTGTTCCT
CAGTTCAAGTTGGGCACTTACGAGAAGACCGGTCTTGCTAGATTCTAATCAAGAGGATGTCAGAATG
CCATTTGCCTGAGAGATGCAGGCTTCATTTTTGATACTTTTTTATTTGTAACCTATATAGTATAGGATT
TTTTTTGTCATTTTGTTTCTTCTCGTACGAGCTTGCTCCTGATCAGCCTATCTCGCAGCTGATGAATAT
CTTGTGGTAGGGGTTTGGGAAAATCATTCGAGTTTGATGTTTTTCTTGGTATTTCCCACTCCTCTTCAG
AGTACAGAAGATTAAGTGAGACCTTCGTTTGTGCGGATCCCCCACACACCATAGCTTCAAAATGTTT
CTACTCCTTTTTTACTCTTCCAGATTTTCTCGGACTCCGCGCATCGCCGTACCACTTCAAAACACCCAA
GCACAGCATACTAAATTTTCCCTCTTTCTTCCTCTAGGGTGTCGTTAATTACCCGTACTAAAGGTTTGG
AAAAGAAAAAAGAGACCGCCTCGTTTCTTTTTCTTCGTCGAAAAAGGCAATAAAAATTTTTATCACG
TTTCTTTTTCTTGAAATTTTTTTTTTTTAGTTTTTTTCTCTTTCAGTGACCTCCATTGATATTTAAGTTAAT
AAACGGTCTTCAATTTCTCAAGTTTCAGTTTCATTTTTCTTGTTCTATTACAACTTTTTTTACTTCTTGT
TCATTAGAAAGAAAGCATAGCAATCTAATCTAAGGGGCGGTGTTGACAATTAATCATCGGCATAGTA
TATCGGCATAGTATAATACGACAAGGTGAGGAACTAAACCATGGCCAAGTTGACCAGTGCCGTTCCG
GTGCTCACCGCGCGCGACGTCGCCGGAGCGGTCGAGTTCTGGACCGACCGGCTCGGGTTCTCCCGGG
ACTTCGTGGAGGACGACTTCGCCGGTGTGGTCCGGGACGACGTGACCCTGTTCATCAGCGCGGTCCA
GGACCAGGTGGTGCCGGACAACACCCTGGCCTGGGTGTGGGTGCGCGGCCTGGACGAGCTGTACGC
CGAGTGGTCGGAGGTCGTGTCCACGAACTTCCGGGACGCCTCCGGGCCGGCCATGACCGAGATCGGC
GAGCAGCCGTGGGGGCGGGAGTTCGCCCTGCGCGACCCGGCCGGCAACTGCGTGCACTTCGTGGCCG
AGGAGCAGGACTGACACGTCCGACGGCGGCCCACGGGTCCCAGGCCTCGGAGATCCGTCCCCCTTTT
CCTTTGTCGATATCATGTAATTAGTTATGTCACGCTTACATTCACGCCCTCCCCCCACATCCGCTCTAA
CCGAAAAGGAAGGAGTTAGACAACCTGAAGTCTAGGTCCCTATTTATTTTTTTATAGTTATGTTAGTA
TTAAGAACGTTATTTATATTTCAAATTTTTCTTTTTTTTCTGTACAGACGCGTGTACGCATGTAACATT
ATACTGAAAACCTTGCTTGAGAAGGTTTTGGGACGCTCGAAGGCTTTAATTTGCAAGCTGGAGACCA
ACATGTGAGCAAAAGGCCAGCAAAAGGCCAGGAACCGTAAAAAGGCCGCGTTGCTGGCGTTTTTCC
ATAGGCTCCGCCCCCCTGACGAGCATCACAAAAATCGACGCTCAAGTCAGAGGTGGCGAAACCCGA
CAGGACTATAAAGATACCAGGCGTTTCCCCCTGGAAGCTCCCTCGTGCGCTCTCCTGTTCCGACCCTG
CCGCTTACCGGATACCTGTCCGCCTTTCTCCCTTCGGGAAGCGTGGCGCTTTCTCAATGCTCACGCTG
TAGGTATCTCAGTTCGGTGTAGGTCGTTCGCTCCAAGCTGGGCTGTGTGCACGAACCCCCCGTTCAGC
CCGACCGCTGCGCCTTATCCGGTAACTATCGTCTTGAGTCCAACCCGGTAAGACACGACTTATCGCCA
CTGGCAGCAGCCACTGGTAACAGGATTAGCAGAGCGAGGTATGTAGGCGGTGCTACAGAGTTCTTGA
AGTGGTGGCCTAACTACGGCTACACTAGAAGGACAGTATTTGGTATCTGCGCTCTGCTGAAGCCAGT
TACCTTCGGAAAAAGAGTTGGTAGCTCTTGATCCGGCAAACAAACCACCGCTGGTAGCGGTGGTTTT
TTTGTTTGCAAGCAGCAGATTACGCGCAGAAAAAAAGGATCTCAAGAAGATCCTTTGATCTTTTCTA
CGGGGTCTGACGCTCAGTGGAACGAAAACTCACGTTAAGGGATTTTGGTCATGAGATC

Note: The yellow highlight is *cce001a*, and the red is *ECORI* and *XbaI* restriction sites respectively.
